# An Axiom SNP genotyping array for Douglas-fir

**DOI:** 10.1186/s12864-019-6383-9

**Published:** 2020-01-03

**Authors:** Glenn T. Howe, Keith Jayawickrama, Scott E. Kolpak, Jennifer Kling, Matt Trappe, Valerie Hipkins, Terrance Ye, Stephanie Guida, Richard Cronn, Samuel A. Cushman, Susan McEvoy

**Affiliations:** 1 Pacific Northwest Tree Improvement Research Cooperative, Department of Forest Ecosystems and Society, Oregon State University, Corvallis, OR USA; 2 Northwest Tree Improvement Cooperative, Department of Forest Ecosystems and Society, Oregon State University, Corvallis, OR USA; 3 USDA Forest Service, National Forest Genetics Laboratory, Placerville, CA USA; 4 National Center for Genome Resources, Santa Fe, NM USA; 5 USDA Forest Service, Pacific Northwest Research Station, Corvallis, OR USA; 6 USDA Forest Service, Rocky Mountain Research Station, Flagstaff, AZ USA

## Abstract

**Background:**

In forest trees, genetic markers have been used to understand the genetic architecture of natural populations, identify quantitative trait loci, infer gene function, and enhance tree breeding. Recently, new, efficient technologies for genotyping thousands to millions of single nucleotide polymorphisms (SNPs) have finally made large-scale use of genetic markers widely available. These methods will be exceedingly valuable for improving tree breeding and understanding the ecological genetics of Douglas-fir, one of the most economically and ecologically important trees in the world.

**Results:**

We designed SNP assays for 55,766 potential SNPs that were discovered from previous transcriptome sequencing projects. We tested the array on ~ 2300 related and unrelated coastal Douglas-fir trees (*Pseudotsuga menziesii var. menziesii*) from Oregon and Washington, and 13 trees of interior Douglas-fir (*P. menziesii* var. *glauca*). As many as ~ 28 K SNPs were reliably genotyped and polymorphic, depending on the selected SNP call rate. To increase the number of SNPs and improve genome coverage, we developed protocols to ‘rescue’ SNPs that did not pass the default Affymetrix quality control criteria (e.g., 97% SNP call rate). Lowering the SNP call rate threshold from 97 to 60% increased the number of successful SNPs from 20,669 to 28,094. We used a subset of 395 unrelated trees to calculate SNP population genetic statistics for coastal Douglas-fir. Over a range of call rate thresholds (97 to 60%), the median call rate for SNPs in Hardy-Weinberg equilibrium ranged from 99.2 to 99.7%, and the median minor allele frequency ranged from 0.198 to 0.233. The successful SNPs also worked well on interior Douglas-fir.

**Conclusions:**

Based on the original transcriptome assemblies and comparisons to version 1.0 of the Douglas-fir reference genome, we conclude that these SNPs can be used to genotype about 10 K to 15 K loci. The Axiom genotyping array will serve as an excellent foundation for studying the population genomics of Douglas-fir and for implementing genomic selection. We are currently using the array to construct a linkage map and test genomic selection in a three-generation breeding program for coastal Douglas-fir.

## Background

For most applications, single nucleotide polymorphisms (SNPs) have become the marker of choice for genetic studies in a wide array of organisms. In forest trees, they are being used to understand the genetic architecture of natural populations, identify quantitative trait loci in pedigrees or natural populations, infer gene function, and assist tree breeding via parental analysis or genomic selection [1–5]. SNPs are desirable because they are found at a high frequency throughout the genome, codominant, usually biallelic, biochemically simple, and amenable to high-throughput genotyping. However, they also have lower information content than other genetic markers such as simple sequence repeats (SSRs).

High-throughput SNP genotyping is typically accomplished using fixed-arrays (i.e., genotyping arrays or SNP ‘chips’), PCR-based methods, or genotyping-by-sequencing (GBS) [6, 7]. Although the PCR-based methods can be used to genotype hundreds to a few thousand SNPs, fixed arrays and GBS are more cost effective for thousands to millions of SNPs. GBS is particularly desirable for some applications because it has low ‘set-up’ costs, SNP discovery and genotyping may occur simultaneously, per-sample costs are low, and there is little or no ascertainment bias in the SNP data. The main disadvantages of GBS are the higher proportions of missing data (i.e., compared to fixed arrays) and the sophisticated bioinformatics needed to analyze the data. GBS has been used to genotype SNPs in a number of conifer and angiosperm tree species [1, 2, 8–10]. Compared to GBS, the fixed-array platforms are more expensive and time-consuming to develop, but the data are easier to analyze, particularly using platform-specific open-source or commercial software (e.g., [11, 12]). Finally, genotyping arrays are better for repeatedly genotyping a common set of SNPs over time, across experiments, or in different populations.

Conifer genomes pose challenges for some aspects of SNP genotyping. First, conifers are genetically diverse; often with at least one SNP every 50 bp [13, 14]. Although this provides ample opportunities for SNP discovery, non-target SNPs and indels may interfere with probe or primer binding, reducing SNP call rates. Second, nuclear genomes of conifers are large and repetitive. In Douglas-fir, for example, less than 50% of the 16 Gbp genome seems to consist of single-copy sequences (i.e., based on a query sequence length of 32) [15]. Large genomes offer many more opportunities for spurious probe or primer binding, which may lead to uninterpretable results. Finally, because conifer genomes are difficult to assemble, inter-locus variants may be misinterpreted as allelic SNPs during SNP discovery. Nonetheless, the design and evaluation of our Axiom array was facilitated by the release of a draft reference genome (v0.5) in 2015, and a newer assembly (v1.0) in 2017 [15, 16].

The main goal of this project was to develop a large-scale SNP genotyping array for Douglas-fir; primarily for use in breeding programs. Key objectives were to develop a platform that would allow forest geneticists and tree breeders to (1) process samples commercially (i.e., outsource SNP genotyping), (2) genotype thousands to tens of thousands of SNPs, and (3) use readily available software for SNP data analysis.

Two widely used genotyping platforms that meet these objectives are the Illumina Infinium® and Affymetrix/Thermo Fisher Axiom® genotyping arrays. The Infinium array can be used to genotype up to 700 K custom SNPs (Infinium iSelect HTS) and comes with software for data analysis (Genome Studio® Genotyping Module). Its main disadvantages are cost and non-overlap in some SNPs across different manufacturing runs. We previously used transcriptome sequencing to identify 278,979 probable SNPs in ~ 20,000 Douglas-fir genes [17]. We then tested a subset of these SNPs (*N* = 8067) using an Illumina Infinium genotyping array, resulting in 5847 successful SNPs (i.e., polymorphic SNPs that can be reliably assayed) [17]. The Infinium array is highly robust, but costs continue to be high on a per-sample basis [6]. The Infinium array has been used in many other plants and animals, including other tree species such as loblolly pine, black cottonwood, white spruce, Norway spruce, and eucalyptus [3, 18–21].

Here, we report the development of an Axiom array capable of genotyping about 28 K SNPs in Douglas-fir. We chose to develop this new, larger Axiom array to characterize geographic variation and practice genomic selection in Douglas-fir. Within the past few years, Axiom arrays have been developed for many agricultural and horticultural crops, including corn, strawberry, rose, rice, apple, soybean, wheat, peanut, and chickpea [22–30]. Although conifers present challenges because of their large genome sizes, an Axiom array has been described for interior spruce [31].

The specific objectives of this study were to (1) design and test a large-scale Axiom genotyping array in Douglas-fir, (2) characterize the performance of the array and the population genetics of individual SNPs in two populations of coastal Douglas-fir (*Pseudotsuga menziesii var. menziesii*), (3) characterize the SNPs in relation to the Douglas-fir reference genome sequence, and (4) conduct a preliminary test of the array on samples of interior Douglas-fir (*P. menziesii* var*. glauca*).

## Results

### Array performance

**Fig. 1:**
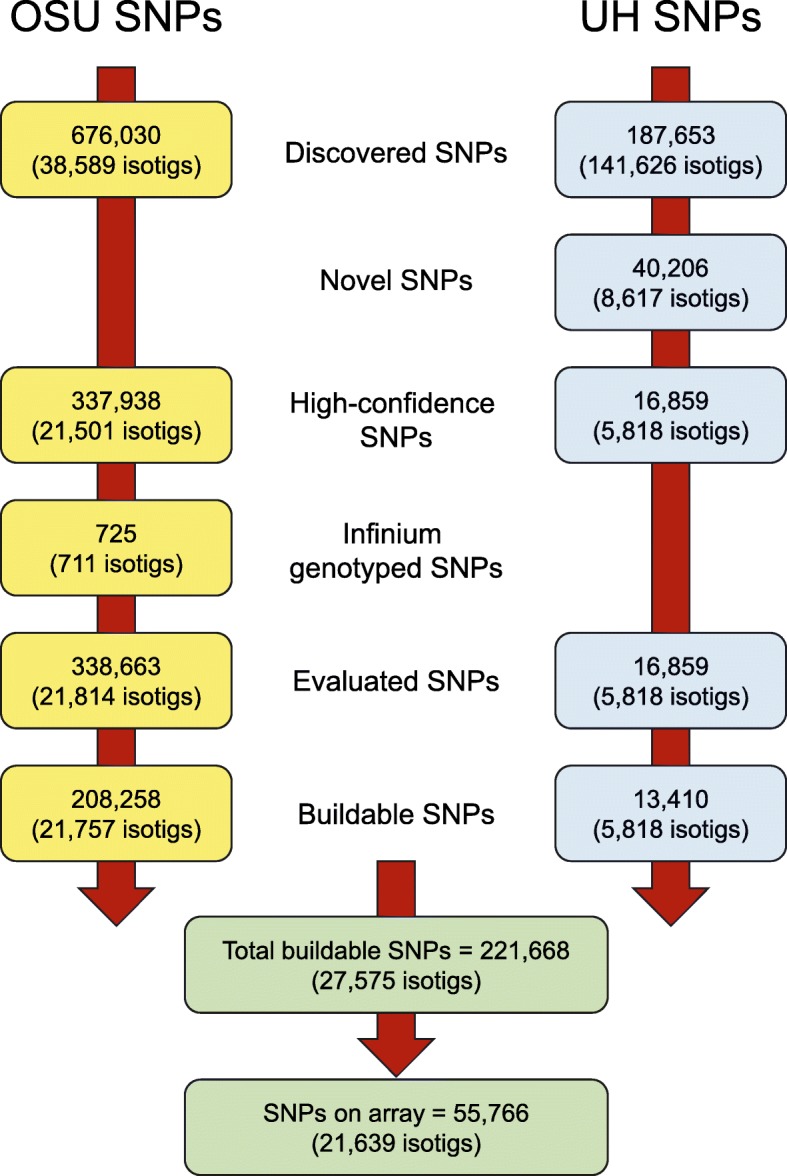
Flow chart of steps used to select SNPs for the Axiom genotyping array. SNPs on the Axiom array were selected from the Oregon State University (OSU) dataset described by Howe et al. [17] and the University of Hohenheim (UH) dataset described by Müller et al. [32]. ‘Discovered SNPs’ are the starting SNPs and isotigs from each dataset. Isotigs are transcript variants assembled using the Newbler de novo assembler. ‘Novel SNPs’ are SNPs in novel UH transcripts, which are transcripts missing from the OSU transcriptome [17]. ‘High-confidence SNPs’ are OSU SNPs with a target SNP probability (*P*_S_) < 0.001 or UH SNPs detected by 2 or 3 SNP detection programs. ‘Infinium genotyped SNPs’ are OSU SNPs previously genotyped using an Infinium genotyping array [17]. ‘Evaluated SNPs’ are the SNPs evaluated for suitability of flanking sequences. ‘Buildable SNPs’ are SNPs with at least one 35-nt flanking sequence with no other (i.e., non-target) high-confidence SNPs or indels. ‘Total buildable SNPs’ are the combined OSU and UH SNPs that were ranked for inclusion on the Axiom array using the variables described in Table 2

We developed and tested an Axiom genotyping array designed to genotype 55,766 SNPs. First, we created a combined dataset of SNPs described by Howe et al. [17] and Müller et al. [32] (i.e., the OSU and UH datasets, Fig. 1). To the OSU dataset of 338,663 SNPs, we added 16,859 UH SNPs that seemed to represent novel transcripts. The combined dataset was filtered using various criteria to arrive at the final set of SNPs tested on the array, which consisted of 52,578 SNPs from the OSU dataset and 3188 SNPs from the UH dataset. Because two assays were included for some SNPs, and the A/T and C/G SNPs required two probesets each, the total number of probesets on the array was 58,350.

The quality control (QC) thresholds used for SNP genotyping affect the number of samples and SNPs for which data are obtained. Thus, we evaluated array performance using three QC approaches (Default, Rescue, and Modified) and five final SNP call rates. The Default protocol used the default Affymetrix QC thresholds (Table S1, Additional file 1) [12]. The Rescue protocols used the default QC thresholds for Phase 1 analysis, followed by a Phase 2 “Rescue” step in which the final SNP call rate was reduced from 97% to as low as 60%. We also tested a Modified QC protocol that was designed to retain more samples by lowering the sample-level and plate-level thresholds in the Phase 1 analysis (Table S1, Additional file 1).

Based on a combined analysis of the first coastal Douglas-fir population (C1) and the interior Douglas-fir population (I1) (*N* = 1920), 1694 samples (88.2%) were successfully genotyped using the Default QC protocol. Because the four Rescue protocols used the same sample-level and plate-level QC thresholds for Phase 1, the number of genotyped samples was the same. When we used the Modified protocol, the number of successfully genotyped samples increased to 1898 (98.9%). For the second coastal Douglas-fir population (C2), 348 of 384 samples (90.6%) were successfully genotyped using the Default and Rescue protocols, and 376 (97.9%) were successfully genotyped using the Modified protocol.

**Fig. 2:**
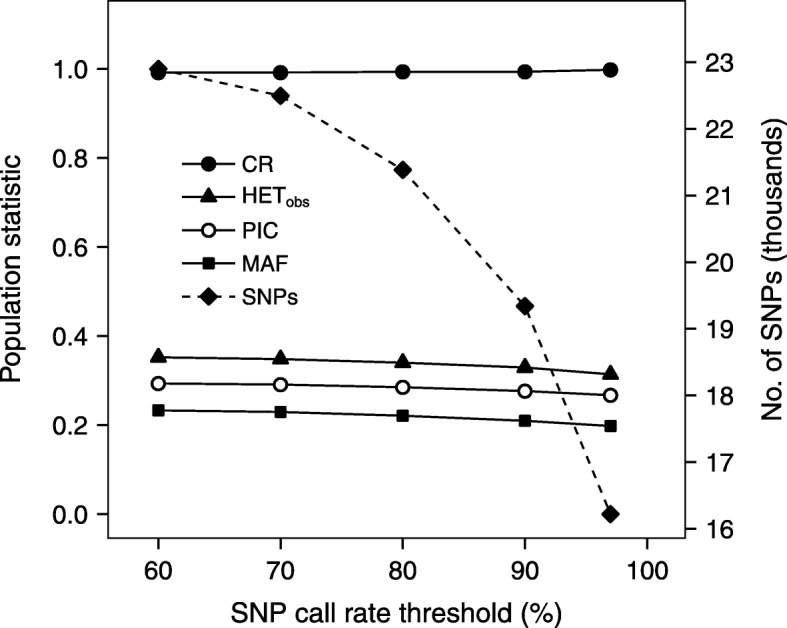
SNP performance and population genetic statistics versus SNP call rate threshold in Douglas-fir. Using all related and unrelated trees in the study, we identified polymorphic SNPs using SNP call rate (CR) thresholds of 60, 70, 80, 90, and 97%. These successful SNPs were then tested on two populations of unrelated trees (N_C1_ = 112 and N_C2_ = 283). The values in the figure are median values averaged across the two populations for SNPs that were polymorphic and in HWE (*P* ≥ 0.01). CR is the measured SNP call rate (percent/100), HET_obs_ is observed heterozygosity, PIC is polymorphic information content, MAF is minor allele frequency, and SNPs are the numbers of polymorphic SNPs in HWE. The scale on the right vertical axis shows the number of SNPs (dashed line), whereas the scale on the left is for all other variables (solid lines)

To assess array performance and repeatability, we assayed SNP success using all samples (i.e., including independent samples from the same tree). Using the Default QC thresholds (with a final SNP call rate threshold of 97%), we were able to genotype 16,177 SNPs in the C1/I1 set of samples and 18,932 SNPs in the C2 population. This is an average of 17,555 SNPs across both populations, and 31.5% of the 55,766 putative SNPs tested on the array (Table 1). We also examined four Rescue protocols, with final SNP call rate cut-offs ranging from 90% down to 60% (Table 1). Averaged across both populations, the number of successful SNPs for the Rescue protocols varied from 20,926 to 25,037 (37.5 to 44.9% conversion). The average number of successful SNPs for the Modified protocol was 22,742 (40.8% conversion; Table S1, see Additional file 1). Each of the analyzed populations (C1/I1 and C2) had successful SNPs that were non-polymorphic in the other population. Thus, if we sum across both populations, the numbers of successful SNPs were considerably higher, ranging from 20,669 for the default QC threshold (97% call rate) to 28,094 for the Rescue protocol using a 60% call rate (37.1 to 50.4% conversion; Table 1). For the Modified protocol, the number of successful SNPs was 25,794 across both populations (46.3% conversion). SNP success was also assayed for two subsets of unrelated coastal Douglas-fir trees (Table S2, see Additional file 1), and results across both populations are shown in Fig. 2. These data were based on 112 unrelated trees from population C1 and 283 trees from C2 analyzed using the Default QC protocol, plus the four Rescue protocols.

**Table 1:** Percentages of successful SNPs using an Axiom genotyping array in Douglas-fir

SNP category^b^	Final SNP call rate threshold^a^					Affymetrix abbreviation [11]
	Default	Rescue				
	97%	90%	80%	70%	60%	
Off-target variant	1	1	1	1	1	OTV
Other	30	29	26	24	23	Other
Call rate below threshold	8	3	2	2	2	CallRateBelowThreshold
Not Converted	40	34	30	27	26	OTV + Other + CallRateBelowThreshold
No minor homozygote	13	13	13	13	13	NoMinorHom
Monomorphic high resolution	16	16	16	16	16	MonoHighResolution
Polymorphic high resolution	31	31	31	31	31	PolyHighResolution
Rescued	–	6	10	13	13	Rescued from Other and CallRateBelowThreshold
Converted^c^	60	66	70	73	74	PolyHighResolution + NoMinorHom + MonoHighResolution + Rescued
Percent successful (population ave)	31.5	37.5	41.6	44.0	44.9	PolyHighResolution + Rescued
Number successful (population ave)	17,555	20,926	23,223	24,548	25,037	PolyHighResolution + Rescued
Percent successful (population sum)	37.1	42.9	46.9	49.5	50.4	PolyHighResolution + Rescued
Number successful (population sum)	20,669	23,917	26,180	27,616	28,094	PolyHighResolution + Rescued

^a^We applied QC thresholds in one or two phases of analysis. The Default protocol consisted of the default Affymetrix parameters, including a CR threshold of 97%. In the Rescue protocols, we used the Default thresholds for phase 1, but then applied lower CR thresholds (60–90%) to the Other and CallRateBelowThreshold categories in phase 2

^b^SNPs (*N* = 55,766) were classified into six categories (OTV, Other, CallRateBelowThreshold, NoMinorHom, MonoHighResolution, PolyHighResolution) and one Rescued category. Successful SNPs were those that were polymorphic with a call rate (CR) exceeding the indicated CR threshold after one or two phases of analysis with alternative quality control (QC) thresholds. Table values are averages from two populations (C1/I1 and C2) that were analyzed separately, except for the ‘population sum’ rows, which are based on sums. The C1/I1 population consisted of coastal Douglas-fir (*N* = 1682) and interior Douglas-fir (*N* = 12) samples that passed QC thresholds and were analyzed together. The C2 population consisted of coastal Douglas-fir (*N* = 348) samples that passed QC thresholds and were analyzed independently

^c^Converted SNPs were those that were successfully assayed using the Default or Rescue protocol, but not necessarily polymorphic

We measured genotyping accuracy using duplicate samples from 58 trees, each genotyped using one to three independent DNA isolations. Excluding missing values, genotyping accuracy was at least 98.4% (i.e., using the Rescue protocol with a final SNP CR of 60%). The inferred allele accuracy for this protocol was 99.2%, with 9.8% missing values. The highest genotyping accuracy was 99.3% for the Default protocol. The inferred allele accuracy for this protocol was 99.6%, with 2.5% missing values.

### Array design variables as predictors of genotyping success

To understand which factors affected probeset success, we first studied whether probeset success was associated with our array design variables (Table 2). Probeset success was 50.0% overall, but higher for selected categories of SNPs and probesets. Not surprisingly, genotyping success was much higher (74.5%) using probesets that targeted SNPs that had already been validated using an Infinium array. Probeset success was associated with other array design variables, but to a lesser extent. Among the four transcript ranking variables, the number of hits to scaffolds was the best predictor of probeset success. Probeset success was 58.5% when the SNP sequence (71 nt) had a single scaffold hit (Table 2). Among the probeset-within-transcript variables, pConvert was most closely associated with probeset success. Probesets with pConvert scores in the upper quartile (Q3) had a probeset success of 57.7% (Table 2). We also derived a final ranking variable that combined the among-transcript and within-transcript information. The best category of this variable (lower quartile; Q1) had a probeset success of 61.5%.

**Table 2:** Transcript and probeset ranking variables versus genotyping success using an Axiom genotyping array

Variable	No. of probesets	Category or mean	Percent or mean		Number	
			Success	Fail	Success	Fail
Transcript ranking variables^a^						
No. of hits to scaffolds^b^ (transcript mean) (v0.5)	58,350	1	58.5	41.5	18,745	13,286
		> 1	41.5	58.5	9403	13,242
		0	27.5	72.5	1011	2663
Transcript confidence score^b^ (absent for UH SNPs)	54,625	Higher	55.8	44.2	13,987	11,087
		Lower	49.6	50.4	14,663	14,888
No. of SNPs per transcript^c^	58,350	**Mean**	**12.00**	**10.36**	**29,159**	**29,191**
		Q3	56.2	43.8	9202	7173
		Q1	43.5	56.5	7375	9570
Combined rank^c^ (transcripts)	58,350	**Mean**	**27,252.2**	**31,096.5**	**29,159**	**29,191**
		Q1	52.5	47.5	7659	6930
		Q3	35.7	64.3	5214	9375
Probeset-within-transcript ranking variables						
Infinium success^b,d^	6173	SNP success	74.5	25.5	4598	1575
Probability of flanking SNPs^b,e^	58,350	Low	50.8	49.2	27,732	26,844
		Moderate	37.8	62.2	1427	2347
No. of perfect alleles^b^ (percent identity = 100%)(v0.5)	58,350	1	53.5	46.5	23,916	20,799
		0	39.2	60.8	5042	7810
		2	25.7	74.3	201	582
pConvert^c^	57,381	**Mean**	**0.615**	**0.595**	**28,508**	**28,873**
		Q3	57.7	42.3	8319	6087
		Q1	41.5	58.5	6429	9059
Target SNP probability^b,f^ (OSU SNPs)	53,958	*P* < 0.0001	55.0	45.0	24,600	20,138
		*P* < 0.001	39.7	60.3	3658	5562
Target SNP probability^b^ (UH SNPs)	3725	3 programs	23.3	76.7	128	422
		2 programs	12.0	88.0	381	2794
Final rank^c,g^ (transcripts and probesets-within-transcripts)	58,350	**Mean**	**27,891.8**	**30,457.6**	**29,159**	**29,191**
		Q1	61.5	38.5	8966	5622
		Q3	46.6	53.4	6800	7788
Other variables						
Recommendation^b,h^	57,295	Recommended	54.7	45.3	17,779	14,748
		Neutral	43.2	56.8	10,691	14,078

^a^Transcripts refer to the Newbler isotigs [17] or putative transcripts [32] used for SNP discovery. v0.5 is version 0.5 of the Douglas-fir reference genome. UH SNPs were those detected by Müller et al. [32], whereas OSU SNPs were those detected by Howe et al. [17]

^b^For the categorical variables, percentages and numbers of probesets are reported for each category and means are absent. All differences among categories were highly significant (*P* <  0.0001) using a likelihood ratio chi-square test

^c^For the ranks and continuous variables, means are reported in bold, and percentages and numbers of probesets are reported for the upper (Q3) and lower (Q1) quartiles. Categories are ranked by probeset success. Successful SNPs were those that had a call rate > 60% and were polymorphic. All differences between means were highly significant (*P* <  0.0001) using a T-test (non-rank variables) or a Wilcoxon rank test (Combined rank and Final rank variables)

^d^For SNPs successfully genotyped with the Infinium platform, Axiom probeset success (74.5%) was significantly greater than the overall probeset success rate of 50.0% (*P* <  0.0001)

^e^Low (rank = 1) or moderate (rank = 2) chance of having flanking SNPs or indels

^f^The *P* <  0.001 category indicates that 0.0001 ≤ *P* <  0.001

^g^The final probeset rank was based on the combined transcript rank plus the probeset-within-transcript variables

^h^The Affymetrix Recommendation variable was not used to select probesets because it is a categorical variable derived from pConvert

**Fig. 3:**
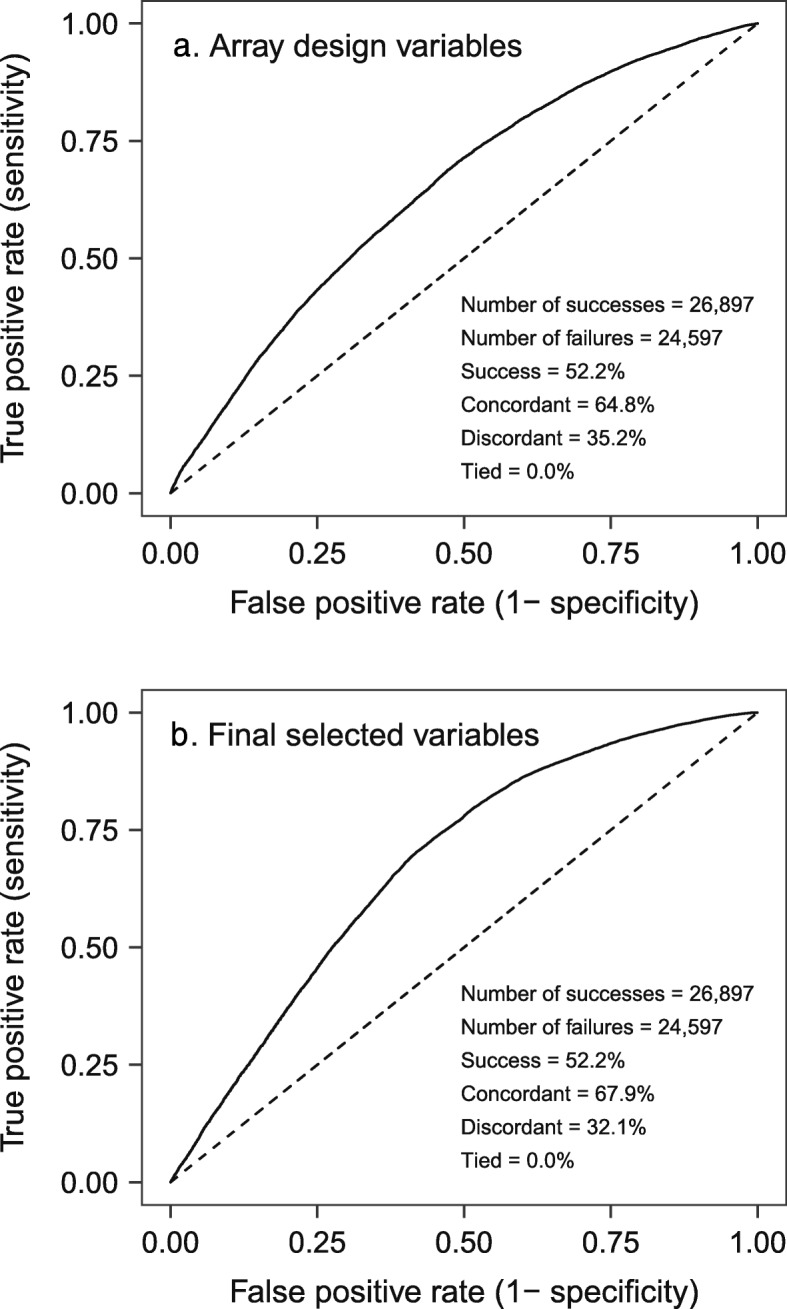
Receiver operating characteristic (ROC) curves for two sets of variables used to predict SNP genotyping success in Douglas-fir. **a** Shows the predictive ability of variables used to design the Axiom array (Table 3). Some of these variables were calculated using an earlier version of the Douglas-fir reference genome (v0.5) [16]. **b** Shows the predictive ability of alternative design variables. We replaced some of the original design variables with new variables calculated using v1.0 of the Douglas-fir reference genome [16], resulting in the final selected variables described in Table 3. ROC curves are used to evaluate binary predictive models (e.g., predictions of SNP success versus failure). Successful SNPs were those that had a call rate > 60% and were polymorphic

Based on logistic regression, the best predictor of probeset success was the number of hits to scaffolds (a transcript ranking variable), followed by pConvert and the target SNP probability (Table 3; columns labeled “Array design variables”). The receiver operating characteristic (ROC) curve for this logistic model is presented in Fig. 3. The ROC curve shows how we can control the accuracy of SNP discovery using logistic regression. Accuracy is measured by plotting the true positive rate (on the Y-axis) versus the false positive rate (on the X-axis). True positive rate is the proportion of real SNPs that are correctly identified. It is also called sensitivity because a highly sensitive SNP classifier would identify most of the real SNPs. The false positive rate is the proportion of false SNPs that are incorrectly classified as SNPs. A highly specific SNP classifier would have a low false positive rate. Using logistic regression, one can choose a SNP probability threshold that meets certain objectives. For example, using the final selected variables (Table 3, Fig. 3) and a predicted SNP probability of 0.5, we could achieve a true positive rate of 76.9% and a false positive rate of 44.9% (Fig. 3, data not shown). That is, we could have refined our set of selected SNPs, identifying almost 80% of the true SNPs, while reducing the false positive rate slightly, from 47.8 to 44.9%. These results suggest our ad hoc approach to SNP selection worked well. However, in the future, we could use our logistic model directly.

**Table 3:** SNP ranking variables versus genotyping success using an Axiom genotyping array and stepwise logistic regression

Variable	DF	Array design variables (ROC area = 0.6449)^a^			Final selected variables (ROC area = 0.6781)^a^		
		Step entered	Chi-square statistic	Chi-square probability	Step entered	Chi-square statistic	Chi-square probability
Scaffold PID (best-hit – second-best hit) (v1.0)^b^	1	–	–	–	1	4557.23	< 0.0001
No. of hits to scaffolds (transcript mean) (v0.5)^c,d^	2	1	1531.38	< 0.0001	–	–	–
Target SNP probability	1	3	642.62	< 0.0001	2	588.16	< 0.0001
pConvert	1	2	730.04	< 0.0001	3	291.26	< 0.0001
Number of perfect alleles (PID = 100%) (v0.5)^c^	2	4	302.18	< 0.0001	–	–	–
Number of SNPs per transcript^d^	66	5	285.60	< 0.0001	–	–	–
Number of hits to singletons (v1.0)^b^	2	–	–	–	4	141.07	< 0.0001
Number of hits to gene models (v1.0)^b^	2	–	–	–	5	85.06	< 0.0001
Number of hits to scaffolds (v1.0)^b^	2	–	–	–	6	31.73	< 0.0001
Probability of flanking SNPs	1	6	43.55	< 0.0001	7	20.08	< 0.0001
Scaffold second-best hit PID (v1.0)^b^	1	–	–	–	8	21.18	< 0.0001
Transcript confidence score	1	7	6.77	0.0093	9	12.91	0.0003
No. of hits to reference transcripts (v1.0)^b^	2	–	–	–	10	14.67	0.0007

^a^Array design variables included variables calculated using v0.5 of the Douglas-fir reference genome. After genotyping, alternative variables were calculated using v1.0 of the reference genome and included in the set of final selected variables. Successful SNPs were those that had a call rate > 60% and were polymorphic. ROC area is the area under the receiver operating characteristic curve using cross-validation

^b^v1.0 variables are the number of BLAST hits or percent identities (PID) using v1.0 of the Douglas-fir reference genome (scaffolds, singletons, gene models, or transcripts) as the target and SNP sequences (71-mers) as the queries

^c^v0.5 variables were calculated using BLAST, Douglas-fir reference scaffolds (v0.5) as the target, and SNP sequences (71-mers) as the queries

^d^Except for ‘reference transcripts,’ ‘transcript’ refers to the Newbler isotigs used for SNP discovery by Howe et al. [17]

### Affymetrix variables as predictors of genotyping success

Affymetrix calculated a Repetitive variable (T, F) based on v0.5 of the Douglas-fir reference genome. We generally excluded repetitive probesets, except 969 probesets for SNPs that had been successfully genotyped using the Infinium array. Of these, 651 (67.2%) were successfully genotyped. After filtering repetitive probesets, array design focused on the pConvert variable. The average pConvert score was slightly higher for the successful probesets (0.615) than for the unsuccessful probesets (0.595) (Table 2). Furthermore, a wide range of pConvert scores was associated with the successful probesets. For example, after excluding the repetitive probesets described above, the pConvert scores for the successful probesets ranged from 0.258 to 0.862, and 38 successful probesets had pConvert scores below the boundary of 0.4 between the ‘neutral’ and ‘not recommended’ categories. For the unsuccessful probesets, the pConvert scores were slightly lower, ranging from 0.106 to 0.832. The Affymetrix Recommendation variable is based on bins of pConvert. We excluded the ‘not possible’ category, and except for the SNPs that had been successfully genotyped using the Infinium array, we also excluded the ‘not recommended’ category. Genotyping success differed between the remaining categories, being 54.7% for the ‘recommended’ category and 43.2% for the ‘neutral’ category (Table 2).

### Genomic context as a predictor of genotyping success

After the array was constructed, we calculated new BLAST variables using an updated version of the reference genome (v1.0). For these SNP-level analyses, the average genotyping success was 50.4%. SNP success was 52.5% for the OSU SNPs (tested SNPs = 52,578) and 14.6% for the UH SNPs (tested SNPs = 3188). For the top category of each BLAST variable, SNP success ranged from 50.9 to 61.0% (Table 4). The best variable was the difference in percent identity (PID) between the best hit and second-best hit. Although we grouped these differences into categories for statistical analysis (Table 4), this difference was 16% PID for the successful SNPs and 11% for the failed SNPs. The number of hits to scaffolds was also a good predictor of SNP success. SNP success was 60.9% for SNPs that had only one hit, 29.1% for SNPs that had more than one hit, and 17.3% for SNPs with no hits. We also conducted logistic regression using selected array design variables plus new variables based on version 1.0 of the reference genome (Table 3). Based on these analyses, the best predictor of SNP success was the difference in PID between the best hit and second-best hit, followed by the target SNP probability and pConvert score (Table 3; Final selected variables). The ROC curve for the logistic model is presented in Fig. 3.

**Table 4:** SNP ranking variables versus SNP genotyping success using an Axiom genotyping array

Variable^a^	No. of SNPs	Category	Percent		Number	
			Success	Fail	Success	Fail
Percent identity (PID)^b,c^						
Scaffold PID (best hit)	55,766	> 80	50.9	49.1	27,936	26,906
		≤ 80	17.1	82.9	158	766
Scaffold PID (second-best hit)	55,766	≤ 80	59.9	40.1	22,775	15,218
		> 80	29.9	70.1	5319	12,454
Scaffold PID (best-hit, second-best hit)	55,766	> 80, ≤ 80	61.0	39.0	22,617	14,452
		> 80, > 80	29.9	70.1	5319	12,454
		≤ 80, ≤ 80	17.1	82.9	158	766
Number of hits^b^						
Number of hits to scaffolds	55,766	1	60.9	39.1	22,946	14,753
		> 1	29.1	70.9	4980	12,115
		0	17.3	82.7	168	804
Number of hits to singletons	55,766	0	51.5	48.5	27,922	26,319
		1	11.8	88.2	79	589
		> 1	10.9	89.1	93	764
Number of hits to gene models	55,766	1	55.8	44.2	10,760	8522
		0	50.8	49.2	16,208	15,705
		> 1	24.6	75.4	1126	3445
Number of hits to reference transcripts	55,766	1	54.1	45.9	12,389	10,529
		> 1	47.9	52.1	3618	3943
		0	47.8	52.2	12,087	13,200

^a^SNP variables are the numbers of BLAST hits or percent identities (PID) using v1.0 of the Douglas-fir reference genome (scaffolds, singletons, gene models, or transcripts) as the target and SNP sequences (71-mers) as the queries. Percentages and numbers of SNPs are reported for each category. Successful SNPs were those that had a call rate > 60% and were polymorphic

^b^All differences among categories were highly significant (*P* < 0.0001) using a likelihood ratio chi-square test

^c^SNP blast hits were categorized as either > 80% or ≤ 80% identity (PID)

### Genomic distributions of SNPs

Based on the transcriptome assemblies used for SNP discovery [17, 32], successful SNPs were associated with 15,038 putative transcripts (isotigs). We also evaluated genome coverage by counting the number of best hits for scaffolds, singletons, gene models, and transcripts using version 1.0 of the reference genome. Of the 28,094 successful SNPs, 27,936 had matches to v1.0 of the reference genome. These 27,936 successful SNPs were associated with 10,428 scaffolds, 181 singletons, 7159 gene models, and 9852 transcripts. Of the 10,428 scaffolds with SNPs, 3744 had a single SNP and 6684 had more than one SNP. For the latter group, the average distance between adjacent SNPs was 52,043 nt.

### Population genetic statistics and effects of QC thresholds

**Fig. 4:**
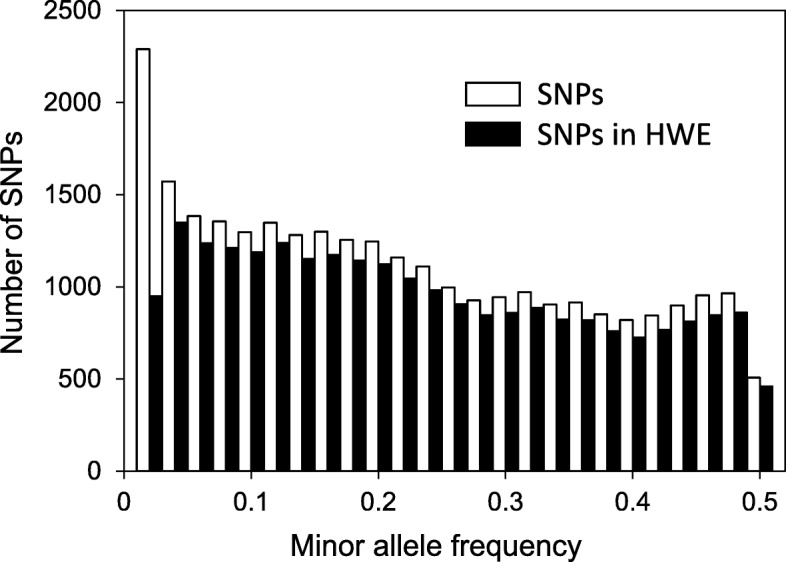
Distributions of minor allele frequencies for successful Douglas-fir SNPs. Open bars represent successful SNPs, whereas solid bars represent successful SNPs that were in Hardy-Weinberg Equilibrium (HWE; *P* ≥ 0.01). Successful SNPs were SNPs that were polymorphic and had SNP call rates > 60%. Minor allele frequencies are averaged across two populations of unrelated trees (C1 = 112 trees and C2 = 283 trees)

Population genetic statistics for SNPs that were successfully genotyped and in Hardy-Weinberg equilibrium (HWE; *P* ≥ 0.01) are reported in Fig. 2 and Table S2 (see Additional file 1). These data were based on the unrelated trees from the coastal Douglas-fir populations (C1 and C2, described in Methods) using the Default QC protocol plus the four Rescue protocols. The statistics differed little between the C1 and C2 populations (data not shown), but there was a very slight decrease in SNP diversity as the CR threshold was increased from 60 to 97% (Fig. 2). Across both populations, for example, median MAF was 0.233 and HET_obs_ was 0.352 using a call rate threshold of 60%, but these values decreased to 0.198 and 0.314 using the Default QC protocol with a call rate of 97% (Table S2, Additional file 1). Similar trends were seen for HET_exp_ and PIC. Because of SNP selection, the distribution of MAF was quite flat (Fig. 4).

## Discussion

We designed and tested an Axiom genotyping array for Douglas-fir. The array included SNP assays for 55,766 potential SNPs that were discovered from transcriptome sequencing projects described by Müller et al. [32] and Howe et al. [17]. Because the SNPs were derived from transcriptome sequences, the array targets SNPs in the expressed genes of the Douglas-fir genome. This approach was chosen to obtain good genome coverage and increase the likelihood that SNPs would be linked to quantitative traits. The array was primarily designed to practice genomic selection, but the validated SNPs will also be valuable for population genetic studies, pedigree-based QTL analyses, evaluation of candidate genes, genome-wide association genetics, and tree breeding activities such as verifying breeding materials, managing inbreeding, characterizing mating systems, and measuring pollen contamination in seed orchards. We used an array-based approach, rather than large-scale GBS, because our aim is to transfer these markers to tree breeders. Using the Axiom array, genotyping can be easily outsourced and the data can be analyzed using user-friendly, array-specific software [11, 12].

### Array performance

In this section, we focus on the performance of the array itself, which we evaluated by testing the array on trees of coastal and interior Douglas-fir. Ultimately, we evaluated SNP success based on 2042 samples (88.6%) that passed the default Affymetrix QC thresholds. Across all Douglas-fir samples, as many as ~ 28 K SNPs were successfully genotyped and polymorphic, depending on the SNP call rate threshold. We worked with Affymetrix bioinformaticists to develop protocols to ‘rescue’ SNPs that did not pass the default Affymetrix QC criteria (e.g., 97% SNP call rate). Lowering the call rate threshold from 97 to 60% using the custom R scripts (R script S1, Additional file 1) increased the number of successful SNPs from 20,669 to 28,094, resulting in conversion rates of 37.1 to 50.4%. Based on preliminary analyses, these are enough SNPs to practice genomic selection in a typical Douglas-fir breeding population.

We evaluated SNP call rate thresholds as low as 60% because genomic selection can be effective with substantial amounts of missing data. Using data imputation, for example, Rutkoski et al. [33] concluded that genomic selection was possible with up to 70% missing data. However, even using a call rate threshold of 60%, the average call rate for our successful SNPs was 96.1% (i.e., less than 4% missing data overall). Furthermore, even at the 60% call rate, genotyping accuracy was 98.4%. By including SNPs with modest or low call rates (i.e., below the default call rate threshold of 97%), it may be possible to improve genome coverage, thereby improving the performance of these SNPs for applications such as genetic mapping, genome assembly, parentage assignment, and genomic selection.

The Axiom array is used to measure hybridization intensities between allele-specific probes and target genomic sequences. Statistical algorithms are then used to infer genotypes by clustering the hybridization intensities and classifying the SNPs into one of six classes: *PolyHighResolution*, *MonoHighResolution*, *NoMinorHom*, *CallRateBelowThreshold*, *OTV*, and *Other*. Examples of the six types of clustering patterns are included in Liu et al. [34] and Affymetrix [11]. However, using custom R scripts, we reclassified some SNPs into a seventh *Rescued* class.

SNP calls were made for the *PolyHighResolution*, *Rescued*, *MonoHighResolution*, and *NoMinorHom* classes (i.e., these classes were ‘converted’ into SNP genotypes; Table 1). The *PolyHighResolution* and *Rescued* SNPs were considered ‘successful’ because they were reliably genotyped and polymorphic. Using the default thresholds, the *PolyHighResolution* SNPs had three well-defined clusters representing the diploid genotypes AA, AB, and BB. In contrast, the *Rescued* SNPs were reclassified from the *CallRateBelowThreshold* and *Other* classes by lowering the final SNP call rate and by implementing a more stringent (lower) confidence score threshold. Averaged across the two populations, 31 to 45% of SNPs were successfully genotyped, depending on the final call rate threshold. The three largest remaining classes (i.e., after excluding the successful SNPs) were *Other*, *MonoHighResolution*, and *NoMinorHom*.

The *Other* class consisted of SNPs that could not be grouped into a few discrete clusters and did not fall into any of the other default classes. Using the default QC thresholds, 30% of SNPs were classified as *Other*. This is at least twice that reported in some species of plants and animals [23, 30, 35–37], but may not be unusual for conifers. For example, the *Other* category represented at least 30% of SNPs in lodgepole pine (*Pinus contorta*), loblolly pine (*Pinus taeda*), and interior spruce (*Picea glauca*, *Picea engelmannii*, and their hybrids) (F. Isik and S. Yeaman, pers. comm). The large *Other* class probably results from the large, repetitive genomes of conifers, and the resulting challenges imposed on genome assembly and SNP discovery (see Introduction). Although we reduced this class from 30 to 23% of SNPs using the rescue protocols, this was still the largest class of SNPs that did not convert.

SNPs may occur in the *Other* class because of poor thermodynamics of the probe, non-target hybridization, and SNPs in the flanking region (i.e., SNPs in the probe target sequence). Non-target hybridization can occur when there are other sequences in the genome similar to the probe. We attempted to avoid this by considering two Affymetrix variables during array design. For the most part, we excluded SNPs when the Affymetrix Repetitive variable was ‘T’ (true). That is, when the number of 16-mer hits between the SNP sequence and the reference genome (v0.5) exceeded 300 hits. We also used the Affymetrix pConvert score to help select SNPs. The pConvert value reflects the thermodynamics of the probe and the number of 16-mer matches in the genome. However, we calculated additional BLAST variables that also helped predict SNP success (i.e., using the SNP sequence as query and reference genome v1.0 as target). The best predictor of SNP success was the difference in percent identity between the best hit and second-best hit. This difference, which was 16% for successful SNPs versus 11% for failed SNPs, could be used to increase the number of successful SNPs in new Douglas-fir arrays or other species. The numbers of hits to scaffolds, singletons, gene models, and transcripts of reference genome v1.0 were also significantly related to SNP success, but to a lesser degree. Finally, because the probability of flanking SNPs was significantly related to SNP success, this variable might also be used to reduce the number of SNPs in the *Other* class.

SNPs in the *MonoHighResolution* class (16% of SNPs) were monomorphic. These putative SNPs may have been transcriptome sequencing errors, or real SNPs found in the SNP discovery population [17] that were not segregating in our validation populations. The discovery population consisted of trees sampled across much of the species’ range, but for SNP validation, we used different trees that were less widely distributed. Furthermore, although we included interior Douglas-fir for SNP discovery [17], we included only 13 interior Douglas-fir trees in this study. Monomorphic genotypes can also result from errors during SNP discovery; i.e., by misinterpreting loci as alleles during transcriptome assembly, or by concluding that sequencing errors are true SNPs. In any case, we did not count these monomorphic SNPs as successful because they were uninformative in our validation populations.

The *NoMinorHom* class (13% of SNPs) may consist of SNPs with particularly low MAFs, or SNPs with segregation distortion, such as SNPs linked to recessive genes with deleterious effects. In either case, we did not count these as successful SNPs because they would probably be excluded from most applications. Overall, it should be possible to reduce the proportion of *MonoHighResolution* and *NoMinorHom* SNPs by enhancing the detection of high-MAF SNPs during SNP discovery. For the final selected variables (Table 3), target SNP probability was the second-best predictor of SNP success. Thus, it should be possible to reduce the *MonoHighResolution* and *NoMinorHom* classes by lowering the SNP probability threshold. Given the declining costs of high-throughput sequencing, this can be easily accomplished by increasing read coverage. Variables associated with genomic context helped identify successful SNPs (Table 3). Presumably, this may be partly due to avoidance of transcriptome assembly errors. The availability of improved genome assemblies should help improve SNP discovery in the future.

The *CallRateBelowThreshold* class consisted of SNPs that did not meet the call rate threshold, but did have acceptable cluster properties. Using the rescue protocols, this class was reduced from about 8% of SNPs using the default thresholds (call rate = 97%) to 2% using a final call rate of 60%.

The final class of SNPs, the off-target variants (*OTV*), are SNPs with unexpected, low-intensity clusters that probably resulted from mismatches between the array probe and genomic target sequence [38]. In diploids, the resulting patterns can be interpreted as either AA, AB, BB, and OTV, or AA, BB, and OTV. These *OTV* clusters are often miscalled as heterozygotes [39]. Although it might have been possible to call some of these SNPs using the OTV_Caller function [11], we did not do this because the proportion of *OTV* SNPs was only 1%.

### SNP characteristics

We evaluated SNP markers based on their genome coverage and population genetic statistics. Based on the transcriptome assemblies used for SNP discovery [17, 32], successful SNPs were associated with 15,038 putative transcripts. We also evaluated genome coverage by counting the number of best hits for scaffolds, singletons gene models, and transcripts using v1.0 of the reference genome. Based on these analyses, successful SNPs were associated with 10,428 scaffolds, 181 singletons, 7159 gene models, and 9852 transcripts. Thus, we conclude that we can genotype about 10 K to 15 K gene loci using the Axiom array. Eventually, relationships among these loci will be clarified using linkage mapping and BLAST analyses using updated versions of the reference genome.

We used a subset of 395 unrelated trees to calculate SNP population genetic statistics for coastal Douglas-fir. These analyses included as many 24,744 successful SNPs averaged across the two test populations (60% call rate threshold), 22,896 of which were judged to be in HWE (*P* ≥ 0.01). We also tested HWE using other *P*-values and multiple comparison adjustments, and these results are available in Data file S1 (see Additional file 2). Over a range of call rate thresholds (97 to 60%), the median call rate for SNPs in HWE ranged from 99.2 to 99.8%, and the median MAF ranged from 0.198 to 0.233. Fig. 2 demonstrates that observed heterozygosities were also high and only modestly affected by the call rate threshold. The high diversity of these SNPs makes them particularly desirable for parental assignment and genomic prediction. However, ascertainment bias should be considered when using these SNPs for other purposes. Because the SNPs on the array were highly selected, MAF is inflated compared to a random sample of SNPs.

## Conclusion

The Axiom genotyping array will serve as an excellent foundation for implementing genomic selection in Douglas-fir. Overall, the successful SNPs (~ 28 K) have high call rates, are well distributed across the genome, have high MAFs, and target expressed genes. The biggest hurdle for implementing genomic selection is the per-sample cost, which may exceed the cost of progeny testing by several fold. Thus, reducing the costs of SNP genotyping will be important. Smaller numbers of SNPs will probably be optimal for validating genotypes, assigning parentage, estimating coancestry, tracking inbreeding, analyzing mating systems, and estimating pollen contamination in seed orchards. We currently use simple-sequence repeat markers (SSRs) for these latter purposes [40], but switching to SNPs would facilitate greater automation of genotyping and data analysis. Custom Axiom arrays are available for 300 to millions of SNPs. However, because fewer than 300 SNPs are needed for some applications, it will be valuable to convert some of the Axiom SNP assays to smaller platforms. For example, we developed cost-efficient Sequenom assays for some of these SNPs, and other low-density platforms are available [6].

Our results indicate that SNP pre-screening would be valuable for large genotyping projects (e.g., > 2000 to 4000 samples). Although we demonstrated that improvements in SNP filtering can increase SNP success, the proportion of successful SNPs would increase dramatically by using pre-screening to exclude the unsuccessful SNPs on the final array. This step would be particularly desirable when the number of samples is much larger than the minimum order size, which is currently 480 samples for the Axiom array [41]. For example, if we had genotyped many thousands of trees, we may have reduced costs by manufacturing a second-generation array with less than half the number of SNPs. Furthermore, the costs of SNP validation could also be reduced by pooling samples across species. The minimum order size for Axiom arrays (480 samples) is larger than what should be needed for estimating assay performance (e.g., SNP call rate) and detecting SNPs with MAFs greater than 0.05. For example, only 59 or 90 trees should be needed to detect these SNPs at a success rate of 95% or 99%, respectively. Thus, one could reasonably validate SNPs for five to eight species simultaneously using as few as 480 samples.

## Methods

### Plant materials

SNP genotyping was conducted on two populations of coastal Douglas-fir (C1 and C2) and one population of interior Douglas-fir (I1). C1 consisted of 1825 trees (1907 samples) from two breeding populations managed by the Northwest Tree Improvement Cooperative: Coos Bay Low and South Central Coast. Trees were selected using three cycles of breeding and testing. The first-cycle selections consisted of 61 trees from native stands in coastal southern Oregon, plus 7 of their progeny growing in first-cycle field tests (i.e., 5 open-pollinated trees and 2 controlled-cross progeny). Next, we selected 609 trees from the second-cycle field tests; i.e., controlled-cross progeny of first-cycle selections. Finally, we genotyped 1033 progeny of the second-cycle selections. These trees came from 24 full-sib families that were growing in the greenhouse. Because some inter-generational crosses were used, the resulting pedigree was complex. In addition to these pedigreed trees, we genotyped 59 trees from a single woodsrun seedlot and 56 trees of uncertain parentage. Because the duplicated tree samples were handled separately (i.e., independent DNA isolations and SNP genotyping), these samples were included in analyses designed to test array performance. However, we used 112 unrelated trees (one sample per tree) to calculate population genetic statistics. Some of the unrelated C1 trees were derived from crosses between trees collected from different geographic locations. C2 consisted of 384 coastal Douglas-fir trees from western Oregon and Washington (one sample per tree). We used these trees to help judge the performance of the Axiom array, and then selected 283 unrelated trees for calculating population genetic statistics. The I1 samples consisted of foliage collected from 13 trees in native stands. All of the genotyped trees described above came from the same broad areas that were sampled for SNP discovery [17, 32]. For each C1, C2, and I1 sample, 10–15 young needles were placed in vials with granular silica gel desiccant (Activa Flower Drying Art), and then stored at room temperature. Once dry, 3 needles were cut into ~ 2 mm lengths, placed in 96-well plates, and then stored at − 20 °C.

### DNA isolation

Dry needles were pre-treated with liquid nitrogen, and then pulverized in a shaker with tungsten beads for two cycles of 60s at 20 Hz. DNA was isolated using the DNeasy-96 Plant Kit (Qiagen), with the addition of a proteinase-K treatment. DNA concentrations were measured using the Pico Green fluorescent dye (Invitrogen) and a Gemini XPS microplate reader (Molecular Devices). Samples with concentrations ≥ 20 ng·μl^− 1^ were used for SNP genotyping.

### Selection of SNPs for the Axiom array

SNPs tested on the Axiom array were derived from the Oregon State University (OSU) dataset described by Howe et al. [17] and the University of Hohenheim (UH) dataset described by Müller et al. [32]. The OSU discovery panel consisted of coastal Douglas-fir trees sampled across Oregon and Washington, plus interior Douglas-fir trees collected across much of its range [17]. The UH discovery panel consisted of Douglas-fir trees from British Colombia, Washington, Colorado, and New Mexico [32]. For the OSU SNPs, we used SNP probabilities and past genotyping success to select SNPs for the Axiom array. *P*_S_ and *P*_F_ are the *p*-values associated with a SNP being a true target SNP or a true variant in the SNP flanking region (i.e., SNP or indel). These were calculated using the methods described by Wei et al. [42], using a MAF value of 0.01 and sequence error rate of 0.01 [17]. From the OSU dataset of 676,030 SNPs, we selected a total of 338,663 SNPs to be evaluated for inclusion on the Axiom array (Fig. 1). Of these, 337,938 were selected because they were detected using a target SNP probability (*P*_S_) of 0.001 (high-confidence SNPs; Fig. 1). Although they had higher *p*-values, we added another 725 SNPs because they had been successfully genotyped using the Infinium platform [17]. Overall, the evaluated dataset included 5847 SNPs that had been previously genotyped using the Infinium array. Of these SNPs, we identified 208,258 that were ‘buildable’; i.e., had a least one 35-nt flanking sequence with no other SNPs or indels using a flanking SNP probability (*P*_F_) of 0.001. To this dataset, we added 13,410 buildable SNPs from the UH dataset that were chosen to target transcripts not already represented in the OSU dataset. To identify novel transcripts, we compared 141,626 UH assembled isotigs (excluding singletons [32]) to the OSU reference transcriptome using a BLAST E-value cutoff of 10^− 10^. Isotigs are transcript variants assembled using the Newbler de novo assembler [17, 32]. We identified 63,286 novel isotigs, 8617 of which contained biallelic SNPs (40,206 SNPs). From these, we selected 16,859 high-confidence SNPs that were detected by two or three SNP detection programs [32]. From these, we identified 13,410 SNPs that were ‘buildable;’ i.e., had a least one 35-nt flanking sequence that did not have flanking SNPs detected by two or three SNP detection programs. In total, we sent 221,668 candidate SNPs from the OSU and UH datasets to Affymetrix (now Thermo Fisher Scientific) to be evaluated by their proprietary software (Fig. 1). After the filtering steps described below, we included 55,766 SNPs on the Axiom array.

Each candidate SNP sent to Affymetrix consisted of the target SNP plus two 35-nt flanking sequences (total = 71 nt). For each of the two flanking sequences per SNP (forward and reverse), Affymetrix calculated a Repetitive indicator variable, pConvert score, and a Recommendation. For the Repetitive variable (T, F), Affymetrix counts the number of 16-nt hits between the SNP sequence and the supplied reference genome. Any flanking sequence with more than 300 hits was classified as repetitive (T). Affymetrix used the v0.5 Douglas-fir reference sequence for this analysis (asm-1.scafSeq.fasta, 5/11/2015 [16]). The pConvert score (0–1) reflects the relative probability of probe success based on the thermodynamics of the probe and the number of 16-nt matches to the reference genome. Probesets with a Repetitive score of T were assigned a pConvert score of 0, and higher pConvert scores indicate a greater probability of SNP success. Affymetrix classified probesets as either ‘not possible’ or ‘buildable,’ and then for the buildable probesets, used the pConvert score to classify each probeset as either ‘recommended’ (0.6 ≤ pConvert ≤1.0), ‘neutral’ (0.4 ≤ pConvert < 0.6), or ‘not recommended’ (0 ≤ pConvert < 0.4).

To design the array, we first removed candidate SNPs that had no acceptable probesets. Unacceptable probesets were those with (1) no corresponding 71-nt matches in the reference genome, (2) SNPs or indels in their target sequences (i.e., in the forward or reverse flanking sequences, *P*_F_ = 0.001), or (3) Affymetrix classifications of ‘not recommended’ or ‘not possible.’ However, we did not remove SNPs if they had already been successfully assayed using the Infinium platform [17], as long as they had at least one buildable probeset. Second, we removed most A/T and C/G SNPs because they occupy twice as much room on the array (i.e., require two probesets to assay).

To select probesets for the array, we first ranked transcripts and probesets-within-transcripts based on the various criteria described below. Then, to maximize genome coverage, we selected the best probeset from each transcript. Because the number of SNPs on the array exceeded the number of transcripts, we cycled through the ranked list of transcripts more than once. In general, we selected one probeset per SNP. However, when this was impossible (i.e., when the transcript had too few SNPs), we selected two probesets per SNP to increase the number of transcripts with successful SNP assays. Thus, on the final array, most SNPs were interrogated by the single best forward or reverse probeset using the criteria described below. Ultimately, we included 58,350 probesets representing 55,766 SNPs on the array. The ranking criteria used in this process are described next.

We ranked the OSU transcripts using three variables. The first variable was the number of BLAST hits between the SNP sequence and the reference genome averaged across all SNPs in the transcript. One BLAST hit was counted for each 65-nt match between the 71-nt SNP sequence and the reference genome. The rank order for this variable (best to worst) was 1, > 1, and 0 hits. The second variable was the transcript confidence score described by Howe et al. [17] (lower is better). These confidence scores were previously derived by comparing our Douglas-fir transcripts to a set of white spruce (*Picea glauca*) unigenes [43]. White spruce was chosen because it represents a closely related genus in the Pinaceae, and had a particularly well curated set of transcript sequences available for comparison. Lower confidence scores represent simpler relationships and (hypothetically) greater confidence that the Douglas-fir assembly was correct. The third variable was the number of SNPs per transcript (higher is better). Transcripts with more SNPs were ranked higher because they have more probesets from which to select the best one. The UH transcripts did not have OSU confidence scores because they were identified as part of a separate SNP discovery project [32]. Thus, they were ranked only by the number of BLAST hits and number of SNPs per transcript.

After ranking the transcripts, we ranked probesets-within-transcripts based on five criteria. First, SNPs successfully genotyped with the Infinium platform were ranked at the top (other SNPs had not been tested using Infinium). The second variable reflected the likelihood of having SNPs or indels (variants) in the flanking sequences. This was accomplished by accounting for variants in the flanking sequences at multiple probability levels. For the OSU target SNPs, we used flanking probabilities of 0.1, 0.01, and 0.001 (*P*_F_) to identify low-, medium- and high-confidence SNPs. For the UH dataset, we identified low-, medium- and high-confidence SNPs based on the number of programs used to call a SNP (1, 2, or 3 SNP detection programs [32]). Next, we identified probesets that had no flanking SNPs, even when all possible SNPs were considered (i.e., low-, medium-, and high-confidence SNPs). These probesets were assigned a rank of 1 (highest priority) because they are least likely to have undetected SNPs in the flanking sequence. Then, we repeated this process after excluding the low confidence SNPs (rank = 2), and then after excluding all but the high confidence SNPs (rank = 3). The third variable was the number of perfect SNP alleles (71-nt sequences) found in the reference genome. We used BLAST to compare each of our two SNP alleles to the reference genome, and then counted the number of these alleles that had at least one match (i.e., possible counts are 1, 2, or 0 alleles, in rank order). Because the reference genome is haploid, a count of 1 suggests the SNP occupies a single genome location. A count of 2 indicates the SNP occupies more than one genome location (i.e., one locus for each SNP allele). A count of 0 suggests there are no matching sequences in the genome. This would occur if the reference had a third alternative allele (e.g., for triallelic SNPs), the target sequence spans an intron, or the target sequence is missing from the genome assembly. Counts of 2 or 0 may also occur via misassembly of our transcriptome sequence or reference genome. The fourth and fifth variables were the Affymetrix pConvert score (higher is better) and the probability of the target SNP. These probabilities were based on the OSU target SNP probabilities (*P*_S_ for the OSU SNPs, smaller is better) or the number of programs that were used to call the target SNP (UH SNPs, higher is better).

### Array processing and SNP calling

DNA samples were processed by GeneSeek (Neogen Genomics, Lincoln, NE) using the standard protocol for the Affymetrix Axiom array. Samples from populations C1 and I1 were processed jointly in two batches and then analyzed together. Samples from population C2 were processed and analyzed separately from C1 and I1. Raw SNP data were analyzed using Axiom Analysis Suite v.1.1.0.616 and the Best Practices Workflow (Affymetrix, Santa Clara, CA). We conducted three types of analyses (Default, Rescue, and Modified) using two phases of quality control (QC) filtering. The Default protocol used the Affymetrix diploid (default) QC thresholds [12]. SNPs were filtered using a SNP call rate cutoff (cr-cutoff) ≥ 97%. Samples (trees) were filtered using a Dish-QC threshold (axiom_dishqc_DQC) ≥ 0.82 and a sample call rate (qc_call_rate) ≥ 97%. The sample call rate is the average SNP call rate across all SNPs for a sample. Plates were filtered using a percent of passing samples (plate_qc_percentsamplespassed) ≥ 95% and a plate call rate (plate_qc_averagecallrate) ≥ 98.5%. The plate call rate is the average sample call rate for passing samples on a plate. Using the Default protocol, the Axiom Analysis Suite classifies SNPs into six categories: *OTV*, *Other*, *CallRateBelowThreshold*, *NoMinorHom*, *MonoHighResolution*, and *PolyHighResolution* [11]. SNPs in the *PolyHighResolution* class (polymorphic high-resolution) were considered successful Axiom SNPs. We also used four SNP rescue protocols that employed two phases of SNP filtering. We used the default thresholds in Phase 1, and then re-classified the SNPs in the *Other* and *CallRateBelowThreshold* categories into a *Rescued* category if they passed a (lowered) SNP call rate cut-off of 90, 80, 70, or 60%. In Phase 2, we used the Ps_CallAdjust SNPolisher function to lower the SNP Confidence Score threshold from the default of 0.15 to 0.10 [11]. The more stringent threshold (0.10) increases the number of missing values (no calls), but improves genotyping accuracy. In the Modified protocol, we changed the default thresholds in Phase 1 as follows: SNP call rate cutoff (cr-cutoff) ≥ 95%; sample Dish-QC threshold ≥ 0.50, sample call rate threshold (qc_call_rate) ≥ 80%, plate percent of passing samples (plate_qc_percentsamplespassed) ≥ 80%, and plate call rate threshold (qc_averagecallrate) ≥ 90% [12]. We then used a SNP call rate threshold ≥80% and SNP Confidence Score threshold ≥ 0.10 in Phase 2 [11]. For the Rescue and Modified protocols, SNPs in the *PolyHighResolution* and *Rescued* classes were considered successful SNPs.

### SNP population genetic statistics

We selected 395 unrelated trees from the C1 and C2 populations, and then used the SAS ALLELE procedure (SAS v.9.4; Statistical Analysis System, Cary, NC) to calculate SNP call rate (CR), MAF, observed and expected heterozygosities (HET_obs_, HET_exp_), polymorphic information content (PIC), and probabilities of deviation from HWE using a chi-square goodness-of-fit test. We excluded SNPs that were not in HWE (*P* <  0.01), calculated statistics separately for each population (C1 and C2), and then averaged the values across the two populations. These analyses were conducted using the Default and Rescue protocols (CR = 97, 90, 80, 70, and 60%).

### Genomic locations and annotations of Douglas-fir SNPs

BLASTN [44] was used to determine the genomic locations and annotations of the Douglas-fir SNPs. We ran BLASTN using default parameters, Axiom probeset sequences (71-mers) as the queries, and four reference genome datasets as the targets: psme.transcript.fna (08/25/2015), psme.allgenes.transcripts.fasta (08/22/2016), Psme_v1.0.scaffolds.fasta (11/12/2015), and Psme_v1.0.singletons.fasta (11/12/2015). These files are part of the Douglas-fir reference sequence database v1.0 [16]. Results from the four BLASTN runs were included in Additional file 2.

### Predictors of SNP success

We evaluated two sets of variables as predictors of SNP success: (1) array design variables and (2) variables calculated using v1.0 of the Douglas-fir reference genome. As described above, some of the array design variables were calculated using v0.5 of the reference genome. After a new reference genome became available (v1.0), we calculated new BLAST variables using the same SNP sequences (71-mers) as queries. The draft reference genome (v0.5) had 18.5 M scaffolds, whereas the newer genome assembly (v1.0) had 2.8 M scaffolds [15].

First, we tested for associations between probeset success (i.e., success or failure; *N* = 58,350) and ten array design variables, including four transcript variables, five probeset-within-transcript variables, and one final rank variable. Although they were not used to select SNPs, we also analyzed the ‘recommended’ and ‘neutral’ classes of the Affymetrix Recommendation variable, which are bins of pConvert. Two variables that were approximately normally distributed (No. of SNPs per transcript and pConvert) were analyzed using a T-test and a Satterthwaite adjustment for heterogeneous variances. Two rank variables (Combined rank for transcripts and Final rank) were analyzed using a Wilcoxon rank test with Monte-Carlo estimation of *p*-values in SAS PROC NPAR1WAY. The remaining variables (see Results) were analyzed as categorical variables using a likelihood ratio chi-square test for independence. Probesets were considered a success if they resulted in polymorphic genotypes in either the C1/I1 or C2 population using the Rescue QC protocol and a CR of 60%.

Second, we tested for associations between SNP success (*N* = 55,766) and seven variables calculated using v1.0 of the reference genome. We used BLASTN to compare SNP sequences (71-mers) to v1.0 scaffolds, singletons, gene models and transcripts using a percent identity (PID) cutoff of 80% for missing values (i.e., no hits). The first two variables consisted of the PID of the best scaffold hit and the PID of the second-best scaffold hit. Although these variables were continuous, many SNPs fell into two classes (PID = 100% or PID = 80% for missing values). Therefore, to test for differences in SNP success, we binned the SNPs into two groups (PID >  80% and PID ≤ 80%). We also evaluated a third variable that captured differences in PID between the top two scaffold hits. For these analyses, we compared SNP success among three SNP categories: (1) PID >  80% for the top two scaffold hits, (2) PID >  80% for the top scaffold hit and ≤ 80% for the second-best hit, and (3) PID ≤ 80% for the top two scaffold hits. A likelihood ratio chi-square test was used to compare the successful and failed SNPs for each variable. For the remaining variables, we counted the number of hits at a PID > 90%, binned these numbers into three categories (1, > 1, and 0, in rank order), and then used a likelihood ratio chi-square test to compare the successful and failed SNPs. This analysis was conducted separately for scaffolds, singletons, gene models, and transcripts, resulting in four variables. SNPs were considered a success if they had a least one probeset that was successful using the criteria described above.

After examining the individual variables, we used logistic regression to develop multivariate prediction equations for SNP success. In the first model, we used the seven array design variables as independent variables (i.e., predictors). In the second model, we dropped the number of SNPs per transcript and replaced the two v0.5 reference genome variables with six v1.0 variables. We conducted these analyses using the SAS LOGISTIC procedure, stepwise model selection option, and cross-validation.

## Supplementary information

**Additional file 1: Table S1.** Quality control (QC) thresholds used to identify successfully genotyped samples, plates, and SNPs (PDF). **Table S2.** Population genetic statistics for successful SNPs genotyped in two populations of Douglas-fir trees (PDF). **Text file S1**. Example R script used for the Rescue SNP genotyping protocols.

**Additional file 2: Data S1**. Characteristics of Douglas-fir SNPs successfully genotyped using an Axiom genotyping array.

## Data Availability

Data generated and analyzed during this study are included in this published article and its supplementary information files. The OSU SNPs used to design the Axiom array are archived as NCBI dbSNP database under submitter handle HOWE_OSU, with ss numbers from 523,746,501 to 524,245,331. Other data are available from the corresponding author upon request.

## Abbreviations

C1: Coastal Douglas-fir population #1
C2: Coastal Douglas-fir population #2
*CallRateBelowThreshold*: Call rate below threshold
CR: SNP call rate
GBS: Genotyping-by-sequencing
HET_exp_: Expected heterozygosity
HET_obs_: Observed heterozygosity
HWE: Hardy-Weinberg equilibrium
I1: Interior Douglas-fir population
MAF: Minor allele frequency
*MonoHighResolution*: Monomorphic high-resolution
NCBI: National Center for Biotechnology Information
*NoMinorHom*: No minor homozygote
nt: Nucleotide
OSU: Oregon State University
OTV: Off-target variant
*P*_F_: Flanking SNP probability
PIC: Polymorphic information content
PID: Percent identity
*PolyHighResolution*: Polymorphic high-resolution
*P*_S_: SNP probability
Q1: Lower quartile
Q3: Upper quartile
QC: Quality control
ROC: Receiver operating characteristic
SNP: Single nucleotide polymorphism
SSR: Simple sequence repeat
UH: University of Hohenheim
