# Supplementary material for: An Axiom SNP genotyping array for Douglas-fir
Source: BMC Genomics. 2020 Jan 3;21:9. doi: 10.1186/s12864-019-6383-9 (PMC6942338; doi:10.1186/s12864-019-6383-9)
Supplement: Supplementary file 1 — Additional file 1: Table S1. Quality control (QC) thresholds used to identify successfully genotyped samples, plates, and SNPs (PDF). Table S2. Population genetic statistics for successful SNPs genotyped in two populations of Douglas-fir trees (PDF). Text file S1. Example R script used for the Rescue SNP genotyping protocols. [file 12864_2019_6383_MOESM1_ESM.pdf]

Table S1. Quality control (QC) thresholds used to identify successfully genotyped samples, plates, and SNPs*

	QC thresholds (SC = SNP Confidence ≥ 0.15 or 0.10)							
	Sample QC thresholds†		Plate QC thresholds‡		Phase 1 (SC ≥ 0.15)	Phase 2 (SC ≥ 0.10)	Results¶ (population average)	
Protocol (SNP CR)	Dish QC	Sample CR (%)	Percent passing samples (%)	Plate CR (%)	SNP CR (%)	SNP CR (%)	Sample success (%)	SNP success (%)
Default (97)	0.82	97	95	98.5	97	N/A	89.4	31.5
Rescue (90)	0.82	97	95	98.5	97	90	89.4	37.5
Rescue (80)	0.82	97	95	98.5	97	80	89.4	41.6
Rescue (70)	0.82	97	95	98.5	97	70	89.4	44.0
Rescue (60)	0.82	97	95	98.5	97	60	89.4	44.9
Modified (80)	0.50	80	80	90.0	95	80	98.4	40.8

*Successfully genotyped samples, plates, and SNPs had QC values ≥ to the indicted thresholds.

†Samples (trees) were filtered using the Dish-QC threshold (axiom_dishqc_DQC) and the sample call rate (qc_call_rate) (Affymetrix 2015). The sample call rate is the average SNP call rate across all SNPs for a sample.

‡Plates were filtered using the percent of passing samples (plate_qc_percentsamplespassed) and the plate call rate (plate_qc_averagecallrate). The plate call rate is the average sample call rate for passing samples on a plate (Affymetrix 2015).

§SNP CR thresholds are the values used to identify successfully genotyped SNPs in the Phase 1 or Phase 2 analysis. The Phase 1 thresholds were the Axiom defaults (SNP Confidence ≥ 0.15 and SNP CR ≥ 97%) for the Default and Rescue protocols, and SNP Confidence ≥ 0.15 and SNP CR ≥ 95% for the Modified protocol. The Phase 2 thresholds were SNP Confidence ≥ 0.10 with SNP CR ≥ 60% to 90%.

¶These thresholds resulted in a sample success rate ranging from 89.4% to 98.4%, and a SNP success rate ranging from 31.5% to 44.9% averaged across the C1/I1 and C2 populations. Successful SNPs were those that passed the indicated QC thresholds and were polymorphic (Table 1).

References

Affymetrix. SNPolisher user guide (version 1.5.2). Affymetrix Inc.; 2015.

Table S2. SNP performance and population genetic statistics versus call rate threshold in Douglas-fir. We used all related and unrelated trees in the study to identify polymorphic SNPs using call rate thresholds of 60, 70, 80, 90, and 97% (see Materials and Methods). These successful SNPs were then tested on two populations of unrelated trees (N_{C1} = 112 and N_{C2} = 283), and then classified into SNPs that were non-polymorphic (1 allele), polymorphic but not in Hardy-Weinberg equilibrium (HWE, P < 0.01), and polymorphic and in HWE (P ≥ 0.01). N_{indiv} shows the range in numbers of trees analyzed for each statistic, N_{allele} is the number of alleles, CR_T is the call rate across all trees in the study, CR_U is the call rate in the unrelated trees, MAF is minor allele frequency, HET_{obs} and HET_{exp} are the observed and expected heterozygosities, and PIC is polymorphic information content as measured using SAS Proc Allele (SAS version 9.4). The values are the averages from the two test populations.

	**Non-polymorphic**				**Polymorphic/Non-HWE**				**Polymorphic/HWE**			
**Statistic**	**MIN**	**MEAN**	**MEDIAN**	**MAX**	**MIN**	**MEAN**	**MEDIAN**	**MAX**	**MIN**	**MEAN**	**MEDIAN**	**MAX**
**CR Threshold = 60%**	**N_{SNPs} = 325**				**N_{SNPs} = 1,878**				**N_{SNPs} = 22,897**			
N_{indiv}	195.0	197.5	197.5	197.5	113.0	180.7	191.5	197.5	113.0	189.3	196.0	197.5
N_{allele}	1	1	1	1	2	2	2	2	2	2	2	2
CR_T	0.988	1.000	1.000	1.000	0.682	0.929	0.967	1.000	0.673	0.965	0.992	1.000
CR_U	0.978	1.000	1.000	1.000	0.553	0.908	0.957	1.000	0.558	0.955	0.992	1.000
MAF	0.000	0.000	0.000	0.000	0.006	0.224	0.207	0.500	0.003	0.245	0.233	0.500
HET_{obs}	0.000	0.000	0.000	0.000	0.000	0.255	0.215	0.874	0.006	0.331	0.352	0.618
HET_{exp}	0.000	0.000	0.000	0.000	0.012	0.297	0.321	0.500	0.006	0.333	0.358	0.500
PIC	0.000	0.000	0.000	0.000	0.012	0.238	0.267	0.375	0.006	0.268	0.294	0.375
**CR Threshold = 70%**	**N_{SNPs} = 325**				**N_{SNPs} = 1,776**				**N_{SNPs} = 22,495**			
N_{indiv}	195.0	197.5	197.5	197.5	132.0	183.5	192.5	197.5	129.5	190.2	196.0	197.5
N_{allele}	1	1	1	1	2	2	2	2	2	2	2	2
CR_T	0.988	1.000	1.000	1.000	0.748	0.940	0.972	1.000	0.749	0.969	0.992	1.000
CR_U	0.978	1.000	1.000	1.000	0.661	0.922	0.963	1.000	0.638	0.960	0.992	1.000
MAF	0.000	0.000	0.000	0.000	0.006	0.214	0.190	0.500	0.003	0.243	0.230	0.500
HET_{obs}	0.000	0.000	0.000	0.000	0.000	0.243	0.200	0.874	0.006	0.329	0.348	0.608
HET_{exp}	0.000	0.000	0.000	0.000	0.012	0.288	0.299	0.500	0.006	0.331	0.354	0.500
PIC	0.000	0.000	0.000	0.000	0.012	0.231	0.251	0.375	0.006	0.267	0.291	0.375
**CR Threshold = 80%**	**N_{SNPs} = 325**				**N_{SNPs} = 1,541**				**N_{SNPs} = 21,384**			
N_{indiv}	195.0	197.5	197.5	197.5	149.5	189.0	194.5	197.5	145.5	192.2	196.5	197.5
N_{allele}	1	1	1	1	2	2	2	2	2	2	2	2
CR_T	0.988	1.000	1.000	1.000	0.832	0.963	0.983	1.000	0.828	0.978	0.993	1.000
CR_U	0.978	1.000	1.000	1.000	0.747	0.952	0.979	1.000	0.706	0.971	0.994	1.000
MAF	0.000	0.000	0.000	0.000	0.006	0.191	0.159	0.500	0.003	0.237	0.221	0.500
HET_{obs}	0.000	0.000	0.000	0.000	0.000	0.219	0.170	0.874	0.006	0.324	0.340	0.604
HET_{exp}	0.000	0.000	0.000	0.000	0.012	0.264	0.257	0.500	0.006	0.326	0.344	0.500
PIC	0.000	0.000	0.000	0.000	0.012	0.214	0.219	0.375	0.006	0.263	0.285	0.375
**CR Threshold = 90%**	**N_{SNPs} = 325**				**N_{SNPs} = 1,264**				**N_{SNPs} = 19,339**			
N_{indiv}	195.0	197.5	197.5	197.5	174.0	193.7	196.0	197.5	169.5	194.5	196.5	197.5
N_{allele}	1	1	1	1	2	2	2	2	2	2	2	2
CR_T	0.988	1.000	1.000	1.000	0.913	0.983	0.992	1.000	0.912	0.987	0.996	1.000
CR_U	0.978	1.000	1.000	1.000	0.874	0.977	0.989	1.000	0.836	0.984	0.994	1.000
MAF	0.000	0.000	0.000	0.000	0.006	0.164	0.118	0.500	0.003	0.230	0.210	0.500
HET_{obs}	0.000	0.000	0.000	0.000	0.000	0.191	0.131	0.874	0.006	0.316	0.329	0.598
HET_{exp}	0.000	0.000	0.000	0.000	0.012	0.232	0.200	0.500	0.006	0.318	0.331	0.500
PIC	0.000	0.000	0.000	0.000	0.012	0.191	0.175	0.375	0.006	0.258	0.277	0.375
**CR Threshold = 97%**	**N_{SNPs} = 324**				**N_{SNPs} = 1,001**				**N_{SNPs} = 16,220**			
N_{indiv}	195.0	197.5	197.5	197.5	187.5	196.2	197.0	197.5	187.5	196.2	197.0	197.5
N_{allele}	1	1	1	1	2	2	2	2	2	2	2	2
CR_T	0.988	1.000	1.000	1.000	0.971	0.993	0.996	1.000	0.971	0.994	0.997	1.000
CR_U	0.978	1.000	1.000	1.000	0.938	0.993	0.998	1.000	0.938	0.993	0.998	1.000
MAF	0.000	0.000	0.000	0.000	0.006	0.133	0.083	0.498	0.003	0.221	0.198	0.500
HET_{obs}	0.000	0.000	0.000	0.000	0.000	0.164	0.105	0.866	0.006	0.306	0.314	0.595
HET_{exp}	0.000	0.000	0.000	0.000	0.012	0.196	0.148	0.500	0.006	0.308	0.318	0.500
PIC	0.000	0.000	0.000	0.000	0.012	0.164	0.134	0.375	0.006	0.251	0.267	0.375

Text file S1. Example R script used for the Rescue SNP genotyping protocols.
Written by Glenn Howe and Chad Dow

```
## DF_advanced_workflow_final.r
setwd("T:\\Groups\\PNWTIRC\\Affy\\R_program_archive")

rm(list = ls(all = TRUE)) ## Remove all objects
phase1 <- "phase1" ## Folder name containing the SNPolisher files
phase2 <- "phase2" ## Folder name that will contain the new files
dir.create(file.path(".",phase2), showWarnings=TRUE)
CR <- 80 ## Selected call rate

## Step 1: Execute default Best Practice Workflow (BPW) using Axiom Analysis Suite
##################################################################################
## BPW uses Ps_Metrics, Ps_Classication, Ps_Visualization, and OTV_Caller.
## read in Ps.performance.txt table from default Best Practice Workflow
perf <- read.table(file.path(".",phase1,"SNPolisher","Ps.performance.txt"), sep="\t", header=T, stringsAsFactors=F)
print(noquote(paste("ps.performance.txt =", nrow(perf))))

## Check number of probesets in each category
perf.PolyHigh <- perf[perf$ConversionType == "PolyHighResolution",]
perf.MonoHigh <- perf[perf$ConversionType == "MonoHighResolution",]
perf.NoMinor <- perf[perf$ConversionType == "NoMinorHom",]
perf.OTV <- perf[perf$ConversionType == "OTV",]
perf.crbt <- perf[perf$ConversionType == "CallRateBelowThreshold",]
perf.other <- perf[perf$ConversionType == "Other",]
perf.Hemi <- perf[perf$ConversionType == "Hemizygous",]

print(noquote(paste("PolyHighResolution (probesets) =", nrow(perf.PolyHigh))))
print(noquote(paste("MonoHighResolution (probesets) =", nrow(perf.MonoHigh))))
print(noquote(paste("NoMinorHomozygote (probesets) =", nrow(perf.NoMinor))))
print(noquote(paste("OffTargetVariant (probesets) =", nrow(perf.OTV))))
print(noquote(paste("CallRateBelowThreshold (probesets) =", nrow(perf.crbt))))
print(noquote(paste("Other (probesets) =", nrow(perf.other))))
print(noquote(paste("Hemizygous (probesets) =", nrow(perf.Hemi))))

## Step 2: Combine 'CallRateBelowThreshold' and 'Other'probeset lists
##################################################################################
## Create combined PS list with "Other" and "CRBT"
ps.other.crbt <- append(perf.other[,1], perf.crbt[,1])
write.table(ps.other.crbt, file.path(".",phase2,"other_crbt.ps"), sep="\t", quote=F, row.names=F, col.names="probeset_id")

## Count SNPs in Other + CRBT categories
n.other_crbt <- length(readLines(file.path(".",phase2,"other_crbt.ps"))) - 1
print(noquote(paste("Other + CallRateBelowThreshold =", n.other_crbt)))

## Step 3: Execute Ps_CallAdjust (threshold=0.1) on combined list
##################################################################################
## Execute Ps_CallAdjust and Ps_Metrics
```

```
##
## AxiomGT1 genotype NoCalls are made for samples with Confidence Scores above the Confidence
## Score Threshold (default =0.15). The Confidence Score is essentially 1 minus the posterior
## probability of the point belonging to the assigned genotype cluster. Confidence Scores
## range between zero and one, and lower confidence scores indicate more confident genotype
## calls. If the Confidence Score rises above the Confidence Score Threshold, the genotype
## call for the sample is converted to a NoCall.
##
## Using a lower call rate threshold can lead to lower quality genotypes passing
## through to the downstream analysis. There is a fairly good correlation between call rate
## and overall quality. The advanced workflow includes a lower call rate threshold for
## rescuing certain probesets in the 'Call Rate Below Threshold' and 'Other' categories after
## the probesets in the recommended categories from the default analysis were put aside.
## However, in these cases, we also implement a more stringent confidence score threshold
## since we are concerned about the accuracy of the genotypes for those samples.

library("SNPolisher")
Ps_CallAdjust(
  pidFile=file.path(".",phase2,"other_crbt.ps"),
  callFile=file.path(".",phase1,"AxiomGT1.calls.txt"),
  confidenceFile=file.path(".",phase1,"AxiomGT1.confidences.txt"),
  threshold=0.1, ## This is one of the key SNP thresholds to change from the default of 0.15
  outputFile=file.path(".",phase2,"CallAdjust_0.1_other_crbt.txt")
)
Ps_Metrics(
  pidFile=file.path(".",phase2,"other_crbt.ps"),
  posteriorFile=file.path(".",phase1,"AxiomGT1.snp-posteriors.txt"),
  callFile=file.path(".",phase2,"CallAdjust_0.1_other_crbt.txt"),
  output.metricsFile=file.path(".",phase2,"metrics_CallAdjust_0.1_other_crbt.txt")
)

## Step 4: Rescue lower resolution 3-cluster probesets from above:
##################################################################################
## nClus = 3
## HomRO > 0
## HetSO > 0
## CallRate = CR
## Probeset rescue
adjust.metrics <- read.table(file.path(".",phase2,"metrics_CallAdjust_0.1_other_crbt.txt"), sep="\t", header=T,
stringsAsFactors=F)
probeset.rescue <- adjust.metrics[adjust.metrics$Nclus > 2,]
  print(noquote(paste("No. of rows of adjust.metrics file =", nrow(adjust.metrics))))
  print(noquote(paste("No. of rows of probeset.rescue file (> 2 clusters) =", nrow(probeset.rescue))))

## Filter probeset.rescue file
obsAA <- probeset.rescue[probeset.rescue$n_AA >= 2,]
obsAB <- probeset.rescue[probeset.rescue$n_AB >= 2,]
obsBB <- probeset.rescue[probeset.rescue$n_BB >= 2,]
obs.intersect <- as.data.frame(intersect(obsAA[,1], obsAB[,1]))
```

```
obs.intersect <- as.data.frame(intersect(obs.intersect[,1], obsBB[,1]))
probeset.rescue <- probeset.rescue[probeset.rescue$probeset_id %in% obs.intersect[,1],]
  print(noquote(paste("probeset.rescue (each genotype > 2) =", nrow(probeset.rescue))))
probeset.rescue <- probeset.rescue[probeset.rescue$HomRO > 0,] ## Homozygous Ratio Offset
  print(noquote(paste("probeset.rescue (HomRO > 0) =", nrow(probeset.rescue))))
probeset.rescue <- probeset.rescue[probeset.rescue$HetSO > 0,]  ## Het Strength Offset
  print(noquote(paste("probeset.rescue (HetSO > 0) =", nrow(probeset.rescue))))
probeset.rescue <- probeset.rescue[probeset.rescue$CR >= CR,]
  print(noquote(paste("probeset.rescue (CR >= ",CR,") =", nrow(probeset.rescue))))
write.table(probeset.rescue[,1], file.path(".",phase2,"rescued_probesets.ps"), sep="\t", quote=F, row.names=F,
col.names="probeset_id")

## Evaluate rescued probesets
Ps_Metrics(
  pidFile=file.path(".",phase2,"rescued_probesets.ps"),
  posteriorFile=file.path(".",phase1,"AxiomGT1.snp-posteriors.txt"), ## Uses orginal posteriors file
  callFile=file.path(".",phase2,"CallAdjust_0.1_other_crbt.txt"),
  output.metricsFile=file.path(".",phase2,"metrics_probeset.rescue.txt")
)
```
